# Epilepsy treatment in neuro-oncology: A rationale for drug choice in common clinical scenarios

**DOI:** 10.3389/fphar.2022.991244

**Published:** 2022-10-06

**Authors:** José Manuel Sánchez-Villalobos, Ángel Aledo-Serrano, Irene Villegas-Martínez, Mohd Farooq Shaikh, Miguel Alcaraz

**Affiliations:** ^1^ Department of Neurology, University Hospital Complex of Cartagena, Murcia, Spain; ^2^ Department of Cell Biology and Histology, School of Medicine, Regional Campus of International Excellence, “Campus Mare Nostrum”, IMIB-Arrixaca, University of Murcia, Murcia, Spain; ^3^ Epilepsy Program, Department of Neurology, Ruber International Hospital, Madrid, Spain; ^4^ Neuropharmacology Research Laboratory, Jeffrey Cheah School of Medicine and Health Sciences, Monash University Malaysia, Bandar Sunway, Selangor, Malaysia; ^5^ Department of Radiology and Physical Medicine, School of Medicine, Regional Campus of International Excellence, “Campus Mare Nostrum”, IMIB-Arrixaca, University of Murcia, Murcia, Spain

**Keywords:** antiseizure medication, brain tumor, glial tumor, seizure, sodium channel blockers, precision medicine

## Abstract

Epilepsy represents a challenge in the management of patients with brain tumors. Epileptic seizures are one of the most frequent comorbidities in neuro-oncology and may be the debut symptom of a brain tumor or a complication during its evolution. Epileptogenic mechanisms of brain tumors are not yet fully elucidated, although new factors related to the underlying pathophysiological process with possible treatment implications have been described. In recent years, the development of new anti-seizure medications (ASM), with better pharmacokinetic profiles and fewer side effects, has become a paradigm shift in many clinical scenarios in neuro-oncology, being able, for instance, to adapt epilepsy treatment to specific features of each patient. This is crucial in several situations, such as patients with cognitive/psychiatric comorbidity, pregnancy, or advanced age, among others. In this narrative review, we provide a rationale for decision-making in ASM choice for neuro-oncologic patients, highlighting the strengths and weaknesses of each drug. In addition, according to current literature evidence, we try to answer some of the most frequent questions that arise in daily clinical practice in patients with epilepsy related to brain tumors, such as, which patients are the best candidates for ASM and when to start it, what is the best treatment option for each patient, and what are the major pitfalls to be aware of during follow-up.

## 1 Introduction

Epileptic seizures are one of the most frequent comorbidities in neuro-oncology and can be either the initial symptom of a brain tumor or a complication during its evolution. Epilepsy is more frequent in primary tumors than in brain metastases (Glantz et al., 2000), although the latter represent the most frequent intracranial tumor (Sánchez-Villalobos et al., 2021b). The prevalence of epilepsy also varies among primary neoplasms according to tumor type and grade, being diffuse low-grade gliomas one of the most highly epileptogenic (Glantz et al., 2000; Pallud et al., 2014).

Currently, epilepsy is a major risk factor for long-term disability in patients with brain tumor-related epilepsy (BTRE) (Maschio, 2012). This is not only due to the negative impact of seizures on quality of life (Rudà et al., 2012), but also to the morbidity associated with both somatic and neuropsychiatric side effects of antiseizure medications (ASM) (Kanner, 2016a). To date, the evidence regarding the use of ASM in BTRE patients is limited. It is overall recommended not to use those drugs with a greater enzyme-inducing effect, given the possibility of modifying the metabolism of antineoplastic drugs. The large availability of ASMs increases both the complexity of drug choice and the possibilities for tailoring treatments according to pharmacokinetics, drug-to-drug interactions, or comorbidities profile, among other factors, such as neoplasm type or genetic profile (Beltrán-Corbellini et al., 2022).

In this narrative review, we provide an overview of ASM in neuro-oncology to help with decision-making, focusing on glial tumors and highlighting the strengths and weaknesses of each ASM. In addition, according to the current evidence, this paper assesses some of the most relevant questions that arise in daily clinical practice in patients with BTRE, such as: i) which patients are the best candidates for ASM prescription; ii) when to initiate ASM; or, iii) which is the best treatment option for each patient concerning their comorbidities or clinical profiles.

## 2 Brief summary on molecular factors in epileptogenesis of brain tumors

Epileptogenesis of brain tumors is influenced by many factors, including tumor location, histological characteristics of the neoplasm, changes in neurotransmitter homeostasis and the peritumoral environment, changes in the integrity of the blood-brain barrier, as well as genetic factors (Ertürk Çetin et al., 2017). To date, several biological and molecular factors have been described that could be involved in the epileptogenesis of brain tumors. Some of them are listed below: with respect to glutamate, high concentrations found in the peritumoral environment would contribute to an increased risk of seizure development and recurrence (Rosati et al., 2010; Goldstein and Feyissa, 2018; Neal et al., 2016). In gliomas, this increase in synaptic concentrations is due to changes in glial membrane transporter systems (De Groot et al., 2011). In addition, glutamergic stimulation of N-methyl-d-aspartate (NMDA) and alpha-amino-3-hydroxy-5-methyl-4-isoxazole propionic acid (AMPA) receptors can activate the intracellular signaling pathways of mammalian target of rapamycin (mTOR), AKT and mitogen-activated protein kinase (MAPK), contributing to both cell growth and epilepsy (De Groot et al., 2011; Englot et al., 2016a). GABAergic signaling is also implicated in both tumor growth and paradoxical excitatory effects mediated by alterations in neuronal and tumor cell chloride ion homeostasis related to cotransporter changes (Pallud et al., 2014). Finally, another factor studied focuses on the isocitrate dehydrogenase 1 (IDH1) enzyme. IDH catalyzes the oxidative decarboxylation of isocitrate to α-ketoglutarate, while in its mutated form, it reduces α-ketoglutarate to D-2-hydroxyglutarate (D2HG) (Turkalp et al., 2014). The D2HG product of IDH1mut can increase neuronal activity by mimicking glutamate activity at the NMDA receptor, and IDH1mut gliomas are more likely to cause seizures in patients (Chen et al., 2016). These represent only some of the molecular factors related to epileptogenesis in brain tumors, other factors such as O-6-methylguanine DNA methyltransferase (MGMT), MMP-9, BDNF, p53 and adenosine kinase (ADK) have also been proposed (Goldstein and Feyissa, 2018).

## 3 Treatment indication in brain tumor-related seizures: When to start and stop antiseizure medication

First, in patients with brain tumors who present with a first seizure, even in the absence of pathological findings on electroencephalogram (EEG) or a second seizure, ASM should be initiated, due to the high risk of recurrence (Chen et al., 2018). Second, there is currently sufficient evidence to discourage treatment with ASM in patients with brain tumors who did not present any seizures (Glantz et al., 2000; Englot et al., 2016a; Chen et al., 2018).

Third, regarding the perioperative use of ASM in patients with brain tumors, a recent Cochrane systematic review did not find evidence of the effectiveness of ASMs (Greenhalgh et al., 2020). Nevertheless, the addition of prophylactic ASM is perioperatively recommended in patients with brain tumors undergoing craniotomy. This treatment should be withdrawn 1 week after surgery (Kuijlen et al., 1996; Glantz et al., 2000; Iuchi et al., 2015; Englot et al., 2016a; Pourzitaki et al., 2016).

Finally, there is no current evidence-based recommendation or consensus on the duration of treatment for epilepsy-related to brain tumors (Chen et al., 2018). Among the factors to be considered in this clinical scenario, we suggest: i) optimal seizure control; ii) complete resection (or not) of the tumor; iii) EEG findings; iv) social and working particularities; v) individualized decision in agreement with patient and caregiver.

## 4 Antiseizure medication for brain tumor-related epilepsy

**FIGURE 1:**
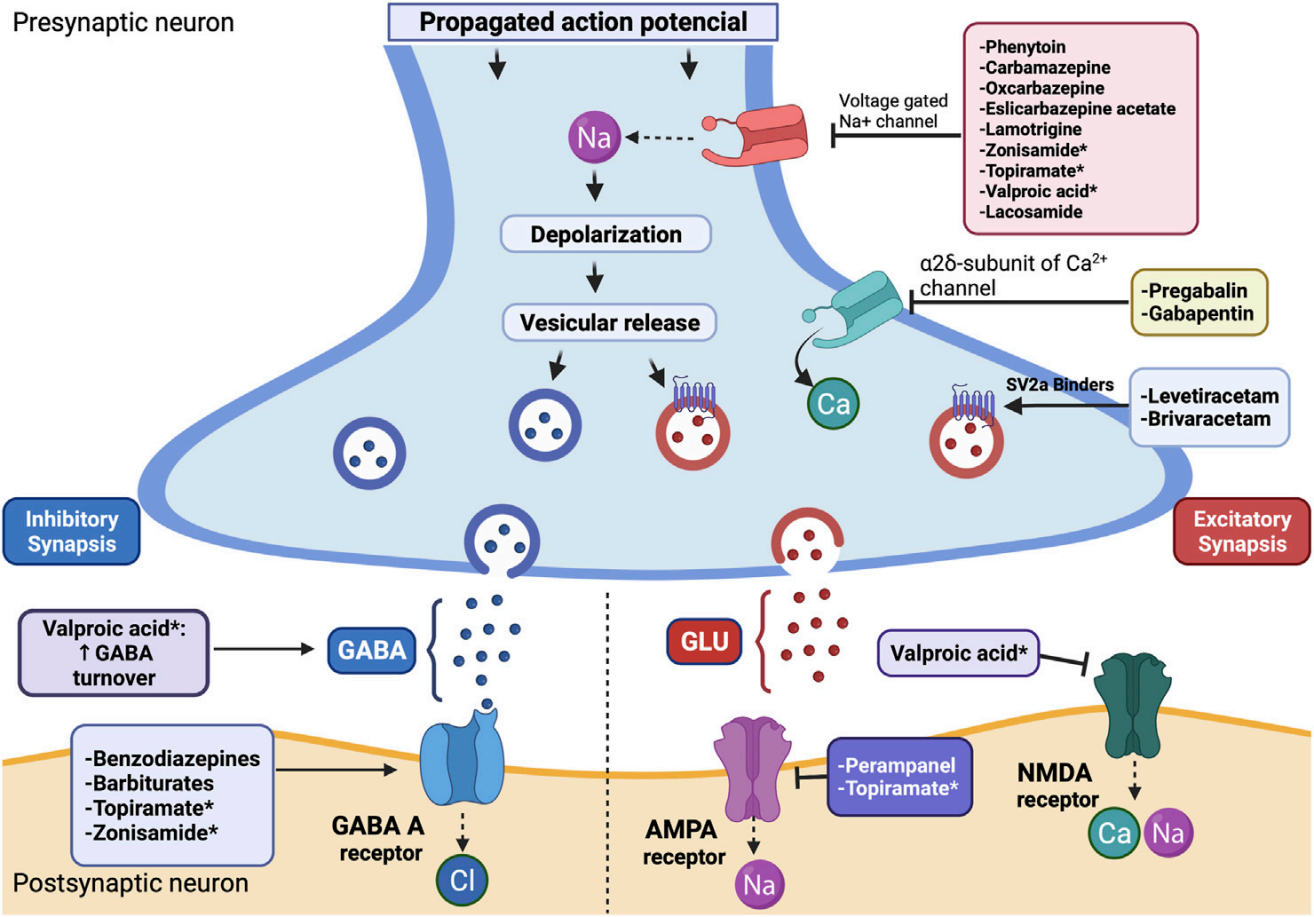
Scheme of the mechanism of action of antiseizure medications. *ASM with more than one proposed mechanism of action. Modified from (Löscher and Klein, 2021). Abbreviations: AMPA, alpha-amino-3-hydroxy-5-methyl-4-isoxazole propionic acid; GABA, gamma-aminobutyric acid; GLU, glutamate; NMDA, N-methyl-d-aspartate.

Currently, the availability of studies evaluating ASM efficacy in patients with BTRE is scarce. Nevertheless, given that epilepsy in these patients is thought to be secondary to a focal brain lesion, usually, the treatment scheme is similar to that of focal-onset epilepsies (Chen et al., 2018). Although the approach to seizures in BTRE patients is multidisciplinary and involves medical, radiotherapeutic, and surgical treatment, in his review we will focus on the use of ASMs. Similarly, although the main target of this article is the control of epilepsy in patients with glial tumors, given that many of the aspects described here are extensive to other lesions, we have considered it necessary to include a comparative table with the main clinical and epidemiological characteristics of the main intracranial lesions (Table 1). For each drug, we will describe the main aspects related to the mechanism of action, pharmacokinetics, main adverse effects, as well as the evidence on the drug in BTRE (Table 2 and Figure 1).

**TABLE 1:** Main characteristics of brain tumors and their relationship with epilepsy.

Types of brain tumors	Age of Debut (years)	Approximate seizure Frequency*	Approx. seizure Freedom Frequency**	Risk Factor for seizures
Glioneural tumors^a, b, c^	15 (DNET), 16–19 (Ganglioglioma)	100% (DNET), 80–90% (Ganglioglioma)	70–90%	Frontotemporal, insular lobe location (Although DNET may be associated with focal cortical dysplasia, the impact of this on epileptogenicity is still unclear).
Low grade glioma^d, e, f^	30–45	60–75%	65–80%	Involvement of the cortex, age below 38 years old, temporal lobe location.
High grade glioma^d, g, h^	60 (Glioblastoma multiforme)	25–60%	40–50% (Glioblastoma multiforme)	Frontal and temporal location.
				Status epilepticus also more frequent in those with frontal or fronto-temporal location.
Brain Metastases^a, d, g, i, j^	>50	20–35%	Variable	Melanoma and lung primary tumor, hemorrhage, supratentorial location, cortical/subcortical involvement
Meningioma^d, k, l^	50–60	20–50%	59–70%	Peritumoral edema on neuroimaging (strongest predictor of seizures), parasagital or convexity tumors, male sex, adults (vs. children).
Primary central nervous system lymphoma^a, g m^	60–70	10–33%	Variable	Cortical involvement

*Percentage of seizure control in patients with preoperative epilepsy. ** Approx. seizure freedom frequency after optimized medical treatment.DNET: Dysembryoplastic neuroepithelial tumor. a (van Breemen et al., 2007), b (Ertürk Çetin et al., 2017), c (Bonney et al., 2016), d (Englot et al., 2016a), e (You et al., 2012), f (Lee et al., 2010), g (Goldstein and Feyissa, 2018), h (Michelucci et al., 2013), i (Singh et al., 2020), j (Wolpert et al., 2020), k (Wirsching et al., 2016), l (Englot et al., 2016b), m (Fox et al., 2019).

**TABLE 2:** Commonly used antiseizure medications in patients with brain tumor related epilepsy.

ASM	Mechanism of action	Drug-to-drug interactions	Strengths	Weaknesses
Levetiracetam	SV2a binder	None	-Pharmacokinetic advantages (rapid and high oral absorption, intravenous formulation, low plasma protein binding, high therapeutic index).	-Psychiatric iatrogenic symptoms (depression, anxiety, psychosis and behavioral disturbances).
			-Wide experience of clinical use.	
			-Potential anti-tumor effect.	-Requires dose adjustment in renal failure and dialysis.
Brivaracetam	SV2a binder	Not clinically significant (Weak inhibition of CYP2C19 *in vitro* studies)	-More selective than levetiracetam for SV2a protein.	-Adjustment required due to liver damage.
			-Rapid crossing of the blood-brain barrier and iv formulation.	
			-Fewer potential psychiatric effects than levetiracetam	-Less clinical experience than levetiracetam.
Lacosamide	SCB (slow inactivation)	None	-Pharmacokinetic strengths (low protein binding, no inhibition or induction of hepatic microsomal isoenzymes of importance, very low potential for drug-to-drug interactions, intravenous use, rapid up-titration).	-Contraindicated in patients with second- and third-degree atrioventricular block
			-Positive effect on neuropathic pain.	
			-No adverse effects in neuropsychiatric sphere.	-Other adverse effects: Dizziness, drowsiness, diplopia.
Carbamazepine	SCB (fast inactivation)	Strong CYP 450 enzyme inducer	-Extensive experience and efficiency in focal epilepsy.	-Potential increase in the metabolism of chemotherapeutic drugs.
			-Positive effect on neuropathic pain.	-Hyponatremia (less than oxcarbazepine and eslicarbazepine acetate).
			-Mood stabilization.	-Osteopenia/osteoporosis.
Oxcarbazepine	SCB (fast inactivation)	Mild enzyme inducer (moderate increase at >900 mg/d)	-Positive effect on neuropathic pain.	-Hyponatremia.
				-Osteopenia/osteoporosis.
			-Mood stabilization.	-No IV formulation
Eslicarbazepine acetate	SCB (slow inactivation)	Mild enzyme inducer	-Single daily dose.	-Hyponatremia
			-Positive effect on neuropathic pain.	
			-Mood stabilization.	-No IV formulation
Lamotrigine	SCB (fast inactivation), calcium channel blocker	None	-Extensive experience and efficiency.	-Allergic skin reactions
			-Mood stabilization.	-Slow up-titration
			-Anti-migraine effect.	-Insomnia
			-Synergism with valproate	-No IV formulation
Valproic acid	SCB, GABA potentiation	Strong enzyme inhibition	-Extensive experience and efficiency.	-High risk of teratogenicity.
			-Mood stabilization.	-Risk of thrombocytopenia/neutropenia (higher thrombocytopenia in those treated with temozolamide).
			-Potential anti-tumor effect.	-Other adverse effects: weight gain, hair loss, hirsutism, and tremor.
Zonisamide	SCB, calcium channel blockade, ↑ GABAr	None	-Single daily dosage.	-Potential negative impact on cognition, weight loss, nephrolithiasis, psychiatric symptoms, metabolic acidosis (Not recommended in patients treated with temozolamide).
			-Anti-migraine effect.	-No IV formulation.
Topiramate	SCB, ↓ AMPA receptors, ↑ GABAr	Mild enzyme inducer (moderate increase at >200 mg/d) Inducer (CYP3A4), inhibitor (CYP2C19)	-Anti-migraine effect.	-Potential negative impact on cognition, weight loss, nephrolithiasis, metabolic acidosis (Not recommended in patients treated with temozolamide).
				-No IV formulation.
Pregabalin/Gabapentine	Calcium channel α2δ-subunit blockers.	None	-Positive effect on neuropathic pain.	-Weight gain.
				-Dizziness and somnolence.
				-Peripheral edema
			-Anxiolytic effect.	-No IV formulation
Perampanel	AMPAr antagonist	Mild enzyme induction (only at high doses)	-Positive impact on sleep architecture.	-Psychiatric symptoms.
			-Potential anti-tumor effect.	-No IV formulation.

AMPA, alpha-amino-3-hydroxy-5-methyl-4-isoxazole propionic acid; CYP, cytochrome; GABA, gamma-aminobutyric acid; NMDA, N-methyl-d-aspartate; SCB, sodium channel blockers. (Kanner, 2016a; Goldstein and Feyissa, 2018; Löscher and Klein, 2021; Kanner and Bicchi, 2022).

### 4.1 Synaptic vesicles protein 2A binders

#### 4.1.1 Levetiracetam

Levetiracetam is an (S)-enantiomer of the ethyl analog of piracetam (Wright et al., 2013). Although the precise mechanism is unknown, in animal models it has been shown to bind to the synaptic vesicle protein 2a (SV2a) (Lynch et al., 2004), an integral transmembrane glycoprotein ubiquitously expressed in all synaptic terminals (Contreras-García et al., 2021).

In pharmacokinetics, most (66%) of levetiracetam is eliminated through the kidneys (Hovinga, 2001). No posology adjustment is needed for patients with hepatic impairment (Wright et al., 2013). Other advantages include rapid and almost complete absorption via oral (96%), low plasma protein binding (<10%), oral and intravenous formulation, and a safety profile with a high therapeutic index and low drug-to-drug interactions (Klitgaard et al., 1998; Wright et al., 2013).

Levetiracetam is frequently prescribed in BTRE patients, being one of the most widely used first-line ASM (Sánchez-Villalobos et al., 2021a). Numerous studies have shown the efficacy of levetiracetam in BTRE patients both in monotherapy (Dinapoli et al., 2009; Merrell et al., 2010; Rosati et al., 2010; Usery et al., 2010; Maschio et al., 2011b; De Groot et al., 2011; Bähr et al., 2012; Rossetti et al., 2014; Berntsson et al., 2018; Cardona et al., 2018; Casas Parera et al., 2019; Kerkhof et al., 2019; Ius et al., 2020; Solomons et al., 2020) and in polytherapy (Wagner et al., 2003; Maschio et al., 2006; Newton et al., 2006; Van Breemen et al., 2009; Haggiagi and Avila, 2019; Chonan et al., 2020; Rudà et al., 2020). In a recent systematic review (Bruin et al., 2021), patients with seizures secondary to grade II-IV gliomas treated with levetiracetam monotherapy had a 6-months seizure freedom rate of 39–96%, with a 6-months failure rate due to adverse effects and ineffectiveness of 1% and 10%, respectively.

As the main side effects, levetiracetam exhibits some relevant downsides, including psychiatric iatrogenic symptoms (7–25%) (Kanner and Bicchi, 2022), such as depression, anxiety, psychosis and behavioral disturbances (Dinkelacker et al., 2003; Dannaram et al., 2012; Lin et al., 2012; Chen et al., 2016; Thelengana et al., 2019). Moreover, patients in treatment with levetiracetam experience more frequent adverse psychiatric effects than those with the other ASMs (Weintraub et al., 2007). In addition, patients with frontal lobe tumors may be at increased risk of neuropsychiatric adverse effects with levetiracetam (Bedetti et al., 2017). Add-on treatment with pyridoxine for the control of levetiracetam-induced behavioral adverse effects might be considered in some patients (Marino et al., 2018; Dreischmeier et al., 2021).

Finally, epigenetic silencing of the MGMT enzyme by levetiracetam could lead to an “antitumor” effect, by increasing the temozolomide efficacy (Bobustuc et al., 2010; Kim et al., 2015). Moreover, a recent study suggests that the use of levetiracetam throughout standard chemoradiation protocol possibly improves the overall survival of patients with isocitrate dehydrogenase (IDH) wild-type glioblastoma (Pallud et al., 2021). However, other previous studies did not show any improvement in the survival of levetiracetam in patients with newly diagnosed glioblastoma (Happold et al., 2016). Thus, further studies are warranted in the future to clarify the potential survival improvement effect.

In summary, levetiracetam has been shown to be a safe and effective drug in BTRE patients, although neuropsychiatric effects should be monitored.

#### 4.1.2 Brivaracetam

Brivaracetam is a selective, reversible, high-affinity ligand of SV2A (15–30 fold higher than levetiracetam) (de Biase et al., 2020). Pharmacokinetically, brivaracetam has the ability to rapidly cross the blood-brain barrier due to its lipophilicity, which is similar to that of benzodiazepines and higher than levetiracetam (Niespodziany et al., 2017). In addition, brivaracetam and levetiracetam, are useful for the treatment of status epilepticus (Santamarina et al., 2019), which makes them both an interesting option in emergency situations. Brivaracetam is extensively metabolized in the liver. Thus, its dose needs to be reduced in patients with liver damage regardless of the Child-Pugh score (de Biase et al., 2020). In contrast, brivaracetam does not induce or inhibit CYP enzymes or the known drug transport system, except for CYP2C19 (weakly inhibited *in vitro* studies). Thus, it has a low potential for clinically relevant drug interactions (de Biase et al., 2020).

To date, Maschio et al. have published the only retrospective study of BTRE-patients treated with brivaracetam as add-on therapy (n = 33). In that study, patients had a high responder rate (78.8%) with a mean follow-up of 10 months. The main cause of drug discontinuation was, again, psychiatric adverse effects (9%) (Maschio et al., 2020a). Although no specific clinical trials comparing psychiatric adverse effects between levetiracetam and brivaracetam are available to date, some studies in non-oncological population show slightly fewer psychiatric adverse effects with brivaracetam (Feyissa, 2019; Villanueva et al., 2019).

Finally, despite being a novel drug, brivaracetam could be considered as an option to be evaluated in BTRE patients, although further studies are needed to unveil both efficacy and tolerability in this population.

### 4.2 Sodium channel blockers

Sodium channel blockers (SCBs) are one of the main families of ASM. We will divide them into different groups: lacosamide, dibenzazepines and lamotrigine.

#### 4.2.1 Lacosamide

Lacosamide is an ASM that selectively increases the slow inactivation of voltage-gated sodium channels, stabilizing the voltage-gated neuronal membranes. In addition, lacosamide appears to interact with collapsing-response mediator protein 2 (CRMP2), thereby enhancing neuronal plasticity (Kellinghaus, 2009). The main pharmacokinetic strengths of lacosamide are low protein binding (less than 15%), no inhibition or induction of several of the hepatic microsomal isoenzymes of importance (CYP2C19 and CYP3A4) to a clinically relevant degree and very low potential for drug-to-drug interactions (Sánchez-Villalobos et al., 2018). Another strength of lacosamide is the possibility of intravenous use in emergency situations requiring rapid uptitration, such as status epilepticus (Strzelczyk et al., 2017). Currently, several studies of lacosamide in polytherapy in BTRE patients have been published (Maschio et al., 2011a; Saria et al., 2013; Villanueva et al., 2016; Maschio et al., 2017b; Rudà et al., 2018; Rudà et al., 2020). The VIBES study, a prospective study (n = 93) that analyzed the efficacy and tolerability of lacosamide as add-on therapy in patients diagnosed of BTRE secondary to low-grade glioma (WHO grade I-II), showed at 6 months a ≥50% reduction in seizure frequency from baseline in 76.7% of patients and being 34.9% seizure-free. 4.3% of patients had drug effects leading to discontinuation (Rudà et al., 2020). Recently, the first retrospective study (n = 132) analyzing the efficacy and tolerability of lacosamide in monotherapy in BTRE has been published, showing absence of seizures in 64.4% of patients after 3 months and 55% after 6 months, with a low dropout rate (1.5%) (Mo et al., 2022).

Regarding adverse effects, these are usually mild and dose-related, sometimes more evident after the morning peak dose, being dizziness and drowsiness the most frequent ones (Mo et al., 2022). On the contrary, it is contraindicated in patients with second and third-degree atrioventricular block. Lacosamide also has proven evidence in treating neuropathic and inflammatory pain in various animal models and observational studies in humans (Stöhr et al., 2006; Alcántara-Montero and Sánchez-Carnerero, 2016; Sánchez-Villalobos et al., 2018), while in the psychiatric sphere, it behaves as a fairly neutral drug (Kanner, 2016a). Finally, *in vitro* antineoplastic effect of lacosamide and brivaracetam in human glioma cells was recently reported (Rizzo et al., 2017).

#### 4.2.2 Dibenzazepines

There are three available different drugs in the dibenzazepine family, from the oldest to the most recent: carbamazepine, oxcarbazepine and eslicarbazepine acetate.

According to their pharmacodynamics, carbamazepine and oxcarbazepine act by blocking the fast inactivation state of gated sodium channels, while eslicarbazepine acetate blocks sodium channel’s slow inactivation. Regarding pharmacokinetics, the main issue is enzymatic induction, which is less pronounced for oxcarbazepine and eslicarbazepine acetate than for carbamazepine. However, carbamazepine shows lower risk of hyponatremia, and larger antiseizure effectiveness in comparative studies (Aledo-Serrano and Gil-Nagel, 2020). There is previous experience in BTRE-patients treated with carbamazepine (Warnke et al., 1997; Zaatreh et al., 2002; Zaatreh et al., 2003; Wick et al., 2005), oxcarbazepine (Maschio et al., 2009; 2012a) and more recently and to a more limited extent with eslicarbazepine acetate (Leslie et al., 2020; Zoccarato et al., 2021). A remarkable aspect of these drugs is that they can have a positive effect in the psychiatric sphere, for example, as mood stabilizers (Kanner, 2016a). Since carbamazepine, as well as phenytoin, are major enzyme inducers, they would not be recommended as first-line treatment in BTRE-patients.

#### 4.2.3 Lamotrigine

Lamotrigine is a first-line ASM for the treatment of focal epilepsy, without enzyme induction features (Perucca and Tomson, 2011). Among its main disadvantages, the need for slow titration and the risk of allergic reactions, mainly skin-related but potentially severe, are of notice, along with the interaction with valproate, which may influence a rigorous dose monitoring (Bruin et al., 2021). This may make lamotrigine an unsuitable starting option in BTRE-patients who needs rapid treatment. With good pharmacokinetics and adverse effects profile, lamotrigine might be a good option in other clinical scenarios.

### 4.3 Valproic acid

The mechanisms of action of valproic acid are not yet fully understood, but its effect on the synthesis and release of γ-aminobutyric acid (GABA) is important, as it increases the effect of GABA in certain brain regions. In addition, the effect on the N-methyl-d-aspartate (NMDA) receptor appears to play an important role in its anti-seizure effect. Pharmacokinetically, the oral bioavailability rate of valproate is close to 100% and approximately 85–95% of the absorbed valproate dose is bound to plasma proteins. In patients with renal insufficiency, chronic hepatic insufficiency or elderly patients, the protein-bound portion is reduced (Baumgartner and Elger, 2020). One of the main problems with valproate is that it inhibits multiple components of CYP system. This might lead to decreased metabolism of some chemotherapeutic agents, increasing their toxicity. Regarding the current evidence, valproate is one of the most historically prescribed ASMs in epilepsy. It is a broad-spectrum ASM that has been used for decades. It is effective in the treatment of focal epilepsies as well as in all types of generalized epilepsy. Similarly, there is extensive experience with the use of valproate in BTRE-patients, both in monotherapy and in polytherapy (Zaatreh et al., 2002; Zaatreh et al., 2003; Wick et al., 2005; Van Breemen et al., 2009; Simó et al., 2012; You et al., 2012; Kerkhof et al., 2013; Yuan et al., 2013).

The most common side effects include weight gain, gastrointestinal complaints, hair loss, hirsutism, and tremor. However, one of the most relevant is thrombocytopenia (12–18% of treated individuals), with advanced age, female sex and high doses of the drug as main risk factors. In addition, the administration of valproate combined with nitrosoureas, etoposide and cisplatin increases bone marrow toxicity, as well as the combination with temozolomide is associated with an increased risk of thrombocytopenia and neutropenia (Bourg et al., 2001; Simó et al., 2012; Bruna et al., 2013).

Finally, it is noteworthy that valproate is associated with increased survival in several observational studies, when administered during chemoradiation therapy in patients with glioblastoma (Weller et al., 2011; Kerkhof et al., 2013; Krauze et al., 2015). Proposed mechanisms would involve increased bioavailability of temozolamide or the histone deacetylase inhibitory activity of valproate, with subsequent sensitization of glioblastoma cells to chemoradiation (Weller et al., 2011; Krauze et al., 2015). However, recently Happold et al. (2016) performed a pooled analysis of the survival association of ASM use at the initiation of chemoradiotherapy with temozolomide (n = 1.869 within four randomized clinical trials) in newly diagnosed glioblastoma, with no survival improvement among patients treated with valproate (and/or levetiracetam) (Happold et al., 2016).

### 4.4 Others

#### 4.4.1 Calcium channel α2δ-subunit blockers

Pregabalin and gabapentin are α2δ-subunit of calcium channel blockers. Although these drugs were initially used for the treatment of seizures, they are now more commonly used for the treatment of neuropathic pain. Nevertheless, pregabalin could represent a valid alternative as add-on therapy in BTRE patients, especially in those with comorbidities such as neuropathic pain or anxiety (Maschio et al., 2012b; Rossetti et al., 2014).

#### 4.4.2 Perampanel

Perampanel is a highly selective, noncompetitive, alpha-amino-3-hydroxy-5-methyl-4-isoxazole propionic acid (AMPA)-type glutamate receptor antagonist. Although it is a relatively novel ASM, some studies have already demonstrated its efficacy as an add-on therapy in BTRE patients (Vecht et al., 2017; Izumoto et al., 2018; Maschio et al., 2019; Maschio et al., 2020b; Chonan et al., 2020). Perampanel presents weak enzyme induction at high doses and require a single daily dose. Additionally, some studies show a positive impact on sleep architecture, as well as relevant side effects in the neuropsychiatric sphere in a subgroup of patients (Kanner, 2016a; Rocamora et al., 2020). Finally, recently some *in-vitro* studies have shown a pro-apoptotic effect of perampanel in human glioblastoma cell lines when used alone, possibly due to increased GluR2/3 expression, as well as a possible synergistic effect when used in combination with temozolamide (Salmaggi et al., 2021).

#### 4.4.3 Topiramate and zonisamide

Topiramate and zonisamide bind to sodium channels and voltage-sensitive calcium channels. Both have been previously used in BTRE-patients and do not present clinically significant enzyme induction features, being an alternative in this patient population (Maschio et al., 2008, 2017a; Lu et al., 2009). Zonisamide has among its advantages a single daily dosage and minimal drug-drug interaction. Among down-sides for both drugs, intravenous formulation is not available, and they show side effects with potential negative impact on cognition and weight loss (Goldstein and Feyissa, 2018). Finally, these drugs are not recommended in patients with gliobastoma and/or high-grade astrocytoma, given their potential side effect with metabolic acidosis and therefore interaction with temozolamide (Grupo Español de Investigación en Neurooncología, 2021).

## 5 Considerations according to particular situations

### 5.1 Elderly patients

Incidence rate of glioblastoma among elderly patients (aged 70 years or older) is 17.5 per 100,000 person-years, representing a relative risk of 3–4 times compared to young adults (Minniti et al., 2019). Therefore, it is interesting to address some of the particularities of ASM in this population. Aging is accompanied by several physiological changes, which affect both the ASM pharmacokinetic and pharmacodynamic characteristics. On the one hand, since renal clearance decreases with aging, the doses of ASMs should be adjusted with renal function. On the other hand, since liver function progressively decreases with aging, the consequent reduction in serum albumin could lead to an elevation of the free fraction of some ASM, potentially increasing the risk of adverse effects. Therefore, liver function should be closely monitored in the elderly patient treated with ASMs (Italiano and Perucca, 2013). Classical ASMs such as phenytoin, carbamazepine, or phenobarbital with a higher enzyme induction profile could reduce plasma concentrations not only of antineoplastic drugs, but also with other drugs commonly taken by elderly patients, such as anticoagulants, antidepressants or antimicrobials (Seo et al., 2020). Likewise, valproate, with an enzyme inhibitor effect, could increase the serum concentrations of some of them, or in the case of some antineoplastic drugs such as temozolamide, increase the hematological toxicity (Simó et al., 2012). And finally, the impact of the ASM on cognition should also be taken into account with phenytoin, topiramate or zonisamide, being some of the ASM that can produce cognition impairment in elderly patients (Seo et al., 2020).

### 5.2 Epilepsy and pregnancy

The possibility of gestation in a woman with BTRE adds a new dimension to the challenge of choosing and subsequently managing ASMs. The risks associated with the use of ASMs during pregnancy are a major concern for all women of childbearing age with epilepsy. Indeed, both the potential adverse effects of ASMs on fetus development, and the effects of uncontrolled seizures on fetus and mother must be considered. In this scenario, seizure control prior to pregnancy represents the most important factor in predicting seizure control during pregnancy (Tomson et al., 2019). Valproate is associated with the highest risks of malformations, as well as adverse cognitive and behavioral outcomes, and should not be used as first line whenever possible in childnearing age women. The risk of major congenital malformations is dose-dependent for valproate and is probably also dose-dependent for other ASMs. Topiramate presents intermediate risk of malformation in specific organs. In contrast, lamotrigine and levetiracetam are associated with the lowest risks of malformations (Tomson et al., 2019). Prior to conception, it would be advisable a careful planning, both for the choice of an optimal ASM with little/no teratogenic potential, as well as for its dosage adjustment, and the initiation of folic acid supplementation prior to conception. During pregnancy, if the woman is taking an ASM that presents substantial changes in clearance (e.g. lamotrigine, levetiracetam and oxcarbazepine), monitoring of the drug level during pregnancy is recommended. Finally, several studies showed no adverse effects of breastfeeding when taking ASMs, therefore breastfeeding would be advisable (Tomson et al., 2019).

### 5.3 Neuropsychiatric comorbidities

Neuropsychiatric comorbidity is a particularly relevant aspect for both patients with epilepsy and brain tumors. Previously, a prevalence of neuropsychiatric disorders of 25–50% has been estimated among people with epilepsy (Lin et al., 2012), while a recent meta-analysis evidenced a prevalence of any mood disorder of 38.2% in oncology patients (Mitchell et al., 2011). Other studies have observed that up to two-thirds of patients with cancer and depression concomitantly present with anxiety symptoms (Smith, 2015). In neuro-oncological patients, especially with frontal-located tumors, prefrontal symptoms such as apathy, irritability, behavioral changes, or irascibility, should be closely evaluated. Moreover, neuropsychiatric comorbidity shows a relevant negative impact on the patient quality of life. It may be aggravated by some of the ASM used to treat BTRE-patients. Therefore, the ASM choice is highly impactful in this specific population.

### 5.4 Sudden unexpected death in epilepsy

Another important aspect to consider in BTRE-patients is sudden unexpected death in epilepsy (SUDEP). Nowadays, it is known that people with epilepsy have an increased risk of mortality compared to the general population, being higher in the first years of the disease, especially in those who are not treated with ASM (Hrabok et al., 2021; Kløvgaard et al., 2021). Other aspects that increase the risk of SUDEP are lack of adherence to treatment and poor seizure control, particularly when bilateral tonic-clonic seizures during sleep are present. Close monitoring and sleep video-EEG studies are mandatory to assess this relevant issue (DeGiorgio et al., 2019; Hrabok et al., 2021). Additionally, in patients at high risk of SUDEP, it is advisable to inform and empower both patient and family about the risk factors and ways to prevent it (Gutiérrez-Viedma et al., 2019).

### 5.5 Glioneural tumors

Glioneural neoplasms, such as disembryoplastic neuroepithelial tumors (DNETs) and gangliogliomas, constitute a specific group of tumors, as they represent highly epileptogenic developmental lesions characterized clinically by early onset of seizures and a tendency to drug resistance (Ertürk Çetin et al., 2017). The frequency of seizures reaches to almost 100% with DNETs and 80–90% with gangliogliomas (van Breemen et al., 2007). They are part of the group of “low-grade epilepsy-associated tumors” (LEATs). LEATs are a specific group of tumors strongly associated with epilepsy. Their characteristics include early-onset drug-resistant epilepsy, slow growth rate, neocortical localization, and temporal lobe predominance (Blümcke et al., 2016). Although DNET may be associated with focal cortical dysplasia, the impact of this on epileptogenicity is still unclear (Bonney et al., 2016). Generally, surgical resection is the corner stone of seizure management for patients with glioneuronal tumors (Krauze et al., 2015). Early surgical intervention and total macroscopic resection represent critically important factors in achieving seizure freedom and thus improving quality of life (Englot et al., 2012b).

## 6 Prognostic factors for seizure control in BTRE patients

There are several factors that may facilitate ASM resistance and prognosis in terms of seizure control. Thus, glioneural tumors (DNET and ganglioglioma) present highest rates of drug resistance. Among the main prognostic factors for seizure control after surgery are shorter duration of epilepsy (less than 1 year) and gross total resection (over subtotal lesionectomy) (Englot et al., 2012). In the case of low grade glial tumors, despite ASMs, approximately one-half of patients may be preoperatively drug-resistant with BTRE. Among some of the factors previously described, insular and/or parietal location of tumor lesions, history of epileptic seizure at diagnosis, and tumor within functional areas are factors associated with drug-resistant seizures (Pallud et al., 2014). Regarding treatment, the extent of resection was associated with improvement in post-treatment seizure control. (You et al., 2012; Pallud et al., 2014). Regarding high-grade gliomas, some works have highlighted that prolonged seizure control is associated with a better Karnofsky performance score, whereas uncontrolled preoperative seizures and parietal lobe involvement would be negative prognostic factors (Kerkhof et al., 2013).

## 7 Conclussion

The choice of the ASM in BTRE-patients is a complex decision determined by many factors. These include pharmacokinetic and pharmacodynamic characteristics, tolerability, efficacy, patient comorbidities, galenic formulations or clinician’s experience, among others. The choice of monotherapy versus polytherapy could be an optimal option to consider, given the minimization of pharmacological interactions. Subsequently, in case of failure to control epilepsy, a rational polytherapy with pharmacodynamic synergies could be an interesting option to consider. In general, ASMs with no (or less) hepatic enzyme induction or inhibition capacity such as levetiracetam, lacosamide, brivaracetam o perampanel would be preferable options to classical ASM given their greater drug-to-drug interactions. Some of the special situations to be considered would be patients with psychiatric comorbidity, elderly patients and women with reproductive desires or pregnancy. Finally, more studies will be needed to establish more optimal decisions on when, with what and until when to maintain ASMs in BTRE-patients.

## Abbreviations

AMPA, alpha-amino-3-hydroxy-5-methyl-4-isoxazole propionic acid; ASM, antiseizure medication; BM, brain metastasis; BTRE, brain metastasis-related epilepsy; DNET, disembryoplastic neuroepithelial tumors; GABA, gamma-aminobutyric acid; IDH, isocitrate dehydrogenase; LEATs, low-grade epilepsy-associated tumors; NMDA, N-methyl-d-aspartate; SCBs, sodium channel blockers; SRS, stereotactic radiosurgery; SV2a, synaptic vesicle protein 2a.
